# Governmental governance of host countries and cross-border merger and acquisition performance: Evidence from listed enterprises in China

**DOI:** 10.1371/journal.pone.0256494

**Published:** 2021-08-23

**Authors:** Kun Liu, Shiyi Wu, Na Guo

**Affiliations:** School of Economics and Management, Fuzhou University, Fuzhou City, Fujian Province, China; Szechenyi Istvan University: Szechenyi Istvan Egyetem, HUNGARY

## Abstract

With globalization, the cases of Chinese enterprises’ cross-border mergers and acquisitions (M&A) are increasing rapidly. The institutional environment of the host country has become an important factor influencing M&A performance, which has a profound impact on the success or failure of cross-border M&A. Based on this, for our study, we selected cases of cross-border M&A of listed companies in China from 2007 to 2018 as research samples to empirically test the impact of the host country’s governance capacity on the cross-border M&A performance of acquirers. It was found that the host country’s governance capacity has a negative effect on the M&A performance in the short term, but in the long term, it can effectively improve the cross-border M&A performance of acquirers. At the same time, specific to the relationship between the governmental governance capacity of six different dimensions and long-term M&A performance, the government effectiveness, regulation quality, and rule of law have the most significant promotional effect on long-term M&A performance. This implies that acquirers should focus on the long-term impact of governmental governance capacity on M&A, and consciously lean toward countries with strong governance capacity in order to obtain long-term value growth when arranging overseas M&A activities. The conclusion of this paper provides a reliable basis on which for companies to achieve sustainable growth in complex economic activities.

## 1. Introduction

In the context of globalization, China’s economy has become deeply integrated into the world economy. Since the corporate “going out” strategy and the “Belt and Road” initiative were formally put forward, China’s foreign direct investment (FDI) has grown rapidly. The foreign investment influence of China has been expanding steadily, and its contribution to the world economy has become increasingly prominent. Cross-border M&A, as one of the crucial ways for Chinese companies to invest abroad, enables companies to obtain natural resources, avoid trade barriers, quickly enter other countries’ markets, and enhance market control and international competitiveness. According to statistics from a PricewaterhouseCoopers report, there were only 126 overseas M&A events of Chinese enterprises, with an M&A transaction value of 10.4 billion US dollars. Since then, cross-border M&A have shown a blowout trend, reaching a peak in 2016, when Chinese companies completed 920 overseas M&A transactions, with a transaction value as high as 202.1 billion US dollars. In the following five years, cross-border M&A transactions declined somewhat, but they are still at an active level. Chinese enterprises are still active overseas buyers in the global market.

Unfortunately, behind the active cross-border transactions, only a few acquirers can achieve their expected strategic goals, and most overseas M&A may cause a loss in corporate value [1, 2], which implies that cross-border M&A are a highly risky activity. Furthermore, the institutional obstacles, cultural conflicts, and economic environment of the host countries in the process of M&A will have a vital impact on the M&A performance [3–5]. For example, Chinalco’s merger with Rio Tinto and CNOOC’s participation in the merger of Chevron and Unocal all ended in failure due to government pressure, which shows that the institutional environment will profoundly influence the success or failure of cross-border M&A. The quality of government is an important factor in determining the inflow of foreign capital. As an important part of the host country’s institutional environment, the governance capacity of a government will profoundly affect the local market investment environment, legal environment, and foreign relations, and will not only affect the location selection of enterprises’ overseas investment but also play a key role in pre-M&A decision-making, valuation during M&A transactions, and post-M&A integration, thus affecting the M&A performance. Therefore, it is necessary to study the influence of the host country’s governmental governance capacity on the overseas M&A performance of Chinese acquirers. In the short term, for the purpose of seeking resources, Chinese acquirers go to countries with backward rule of law and serious corruption to carry out M&A. Such M&A have relatively higher short-term benefits due to obtaining a large amount of resources. However, in the long term, ignoring the negative impact brought about by the governance capacity of the government will increase the risk of cross-border M&A, such as deprivation of their legal property, inability to constrain contracts, and difficulty in obtaining fair competition opportunities. Thus, the value of the company may also be reduced. On the contrary, in countries with a higher level of governance capacity, their policy stability and government management efficiency are higher; therefore, acquirers can obtain higher public service quality and infrastructure after investment, reducing unnecessary rent-seeking costs, and have lower investment risk. In addition, countries with high levels of governmental governance capacity often correspond to effective government supervision and sound legal systems. In such an environment, the acquirer can obtain opportunities for fair competition and reduce the degree of information asymmetry with the target company. The stable and open social environment is also conducive to the integration of resources between the acquirer and the target, which contributes to the long-term value appreciation of the acquirer after the merger.

However, current scholars mainly discuss the relationship between institutional environment or governance capacity and the location of companies’ outbound investment. For example, some scholars believe that a good institutional environment can reduce resource misallocation and non-productive consumption. Therefore, the higher the level of governmental governance in a host country, the more attractive it is to investment from Chinese companies [6, 7]. While some scholars believe that one of the main motives of China’s FDI is to seek natural resources, so regions with poor system quality and high probability of default are more likely to attract Chinese enterprises to invest, and Chinese enterprises’ foreign investment has the characteristics of risk appetite [8–10]. There are also a few scholars who have analyzed the impact of institutional factors on the success or failure of corporate cross-border M&A investments and performance. The indicators for measuring investment effectiveness mainly include, first, taking the completion of M&A as a binary variable to measure the success or failure of investment transactions [11, 12] and, second, using financial indicators such as innovation performance or return on total assets (ROTA) to observe whether corporate performance has improved after M&A [13, 14]. For example [11], Studied 421 M&A in Chinese companies from 2003 to 2016, and the results showed that there was a significant negative correlation between the host country’s institutional risk and the success rate of cross-border M&A [13]. Selected the 2008–2014 Chinese listed companies’ overseas M&A as the research object, and measured the innovation performance by the number of patent applications. The results showed that the development level of a host country institution has a significant positive effect on the innovation performance of overseas M&A [14]. Used Chinese industrial companies that made overseas investments from 2000 to 2007 as a research sample, and found that the host country’s institutional environment has a differential impact on the performance of Chinese foreign-invested companies. The institutional environment of developed countries has a positive effect on corporate performance, while the institutional environment of developing countries has a negative effect.

Based on the existing research, it can be seen that the current scholars’ discussion on institution and investment is still focused on the impact of a one-dimensional institutional environment on the location selection for enterprises’ FDI, and the research has not yet reached a consistent conclusion. Furthermore, a few scholars discuss the cross-border M&A performance mainly based on the system perspective, and only measure the M&A performance by the completion of M&A transactions or financial indicators, without distinguishing short-term and long-term effects, which has the problem of inaccurate measurement of M&A performance. Based on this, from the perspective of governance capacity, we selected 460 cross-border M&A transactions of listed companies in China from 2007 to 2018, measured the governance capacity of the host government by using the World Bank’s worldwide governance indicators (WGI), and deeply analyzed the comprehensive impact of the governance capacity of the host government on the cross-border short-term and long-term M&A performance of acquirers. Furthermore, the WGI includes six specific dimensions: voice and accountability, political stability, government effectiveness, regulatory quality, rule of law, and control of corruption. We also examined the long-term impact of different dimensions of governance capacity on M&A performance. We hope to provide useful suggestions and insights for Chinese enterprises to improve their overseas M&A performance.

## 2. Literature review

### 2.1. Research on cross-border M&A performance

Regarding the research on the M&A performance, most scholars have shown that M&A can bring excess returns to the target company. However, for the performance of acquirers, the current research results are not consistent. One view is that M&A can also bring excess returns to the acquirer [15]. However [16], Studied 937 M&A events during 1955–1987, and found that the average loss of the acquirer reached 10% within five years. Similarly [17], Selected 539 M&A events of American listed companies from 2007 to 2014 as samples, and the research also showed that the excess return loss of the acquirer in the window period was about 2%. With the acceleration of Chinese companies’ "going out" pace, Chinese companies have gradually become a major force in the global M&A wave. Many scholars have also conducted research and discussion on the cross-border M&A performance of Chinese acquirers [18]. Measured the overseas M&A performance of Chinese enterprises by using market model, FF3FM model and event research method and found that the market gave positive evaluation to Chinese enterprises’ cross-border M&A in the short term. At the same time, from the medium and long-term perspective, Chinese acquirers’ overseas M&A have achieved non-negative excess returns on the whole. However [19], and [20] Respectively conducted empirical analysis on the cross-border M&A events of Chinese enterprises at different times, and the results showed that cross-border M&A can bring positive benefits to enterprises, but with the passage of time, the cross-border M&A performance of acquirers declined year by year, which shows that the positive impact of cross-border M&A on Chinese enterprises is limited.

### 2.2. Research on factors influencing the cross-border M&A performance

At present, scholars’ research on the factors affecting the cross-border M&A performance is mainly carried out from the transaction level, corporate level and macro level. At the transaction level [21], thought that because the information of listed companies was more transparent, it can reduce the degree of information asymmetry between the two parties, so compared with non-listed companies, M&A of listed companies can bring higher benefits [22]. Conducted a research on the phenomenon of full cash payment for Chinese companies’ cross-border M&A, and found that cash payment significantly reduced M&A performance, and full cash payment did not send a good signal. At the corporate level, the nature of corporate ownership [2], major shareholder shareholding ratio [23], corporate governance [24] and management characteristics [25] are all important factors that affect M&A performance. For example [24], found that corporate internal governance can effectively alleviate agency conflicts and promote the improvement of corporate cross-border M&A performance. And the better the regional institutional environment of the acquirers, the more significant the impact of internal governance on cross-border M&A performance. At the macro level, the influencing factors discussed by scholars include cultural differences [26] and institutional environment [27, 28]. For example [27], used the economic freedom index and the difficulty of doing business to measure the host country’s control system. The results showed that they were positively and negatively correlated with M&A performance, and the host country regulation system had a significant impact on the cross-border M&A performance of enterprises.

### 2.3. Research on institutional environment and foreign direct investment

At present, the conclusions about the impact of the institutional environment on overseas investment are not uniform. Some scholars believe that a good institutional environment can promote a country’s productive activities and reduce the distortion of resource allocation and non-productive consumption, such as rent-seeking, black market transactions, and corruption, which is conductive to reducing investment costs [6]. Conducted a study on China’s direct investment in ASEAN countries from 2003 to 2015, and the results showed that the host country’s macro-system endowment was positively correlated with China’s foreign investment. The higher the governance level of the host country, the more it can attract investment from China. The research of [7] also showed that companies preferred to conduct M&A in countries with better institutional environments. The possible reason is that there is a certain distance between China’s governance level and developed countries. Chinese companies have therefore accumulated a lot of experience in carrying out value-added activities in a lower governance level environment. Therefore, Chinese enterprises have more advantages in investing in countries and regions with similar or lower governance level than those in developed countries.

However [29], used state-owned enterprises as the research object, and the results showed that the foreign direct investment of state-owned enterprises presented a "risk preference" nature, that is, state-owned enterprises were more inclined to invest in higher-risk areas. In addition, there is a certain substitution relationship between the risk level of the host country and natural resources. Similarly [30], conducted an empirical analysis of China’s OFDI in the belt and road initiative countries and found that the improvement of the quality of government governance in the host country may hinder China’s direct investment in it.

### 2.4. Research on institutional environment and M&A effect

In the existing literature, there are also a few that discuss the impact of institutional factors on the success or failure of corporate cross-border M&A. The indicators for measuring investment effectiveness mainly include: First, taking the completion of M&A as a binary variable to measure the success or failure of investment transactions. Second, using financial indicators such as innovation performance or return on total assets (ROTA) to observe whether corporate performance has improved after M&A.

[11] Conducted a study on 421 M&A events of Chinese enterprises from 2003 to 2016. The results showed that there is a significant negative correlation between the host country’s institutional risk and the success rate of cross-border M&A. [12] found that the host country’s institutional environment and friendly bilateral political relations have a certain complementary effect, which can significantly increase the success rate of Chinese companies’ foreign investment. [13] selected the 2008–2014 Shanghai and Shenzhen listed companies’ overseas M&A as the research object, and measured the innovation performance by the number of patent applications. The results showed that the host country’s system development level has a significant positive effect on the innovation cross-border M&A performance. [14] taking Chinese industrial enterprises that made overseas investment from 2000 to 2007 as research samples, empirically examined the impact of host country’s institutional environment on enterprise performance, and found that the host country’s institutional environment has different effects, while the developed country’s institutional environment has a positive effect on enterprise performance, while the developing country’s institutional environment has a reverse effect.

According to a review of existing research, it can be found that the current research on the institutional environment is still mainly focused on the analysis of the impact of the institution on the location selection of enterprises’ foreign direct investment. Some scholars have conducted research on the institution and M&A performance. However, the research, mainly from the perspective of the institutional environment, selects the economic freedom index and the ease of business index to measure the institutional environment risk, and only measures the M&A effectiveness by observing whether the M&A is completed or changes in some financial indicators. However, the completion of the transaction does not mean that the post-merger integration is smooth or the cross-border transaction can add value to the company. And the financial indicators often contain certain noise information due to the modification of financial data and the great freedom of report making, so it is not accurate to measure the value-added of the enterprise after the merger. Therefore, based on the perspective of governmental governance capacity, we measure M&A performance with event research method, deeply analyze the influence of host government governance capacity on short-term and long-term cross-border M&A performance, and the role of governmental governance capacity in the process of M&A, and then put forward relevant suggestions, which will provide beneficial suggestions and enlightenment for Chinese enterprises’ overseas M&A and government policy making.

## 3. Research hypothesis

Governmental governance capacity includes national policy formulation and implementation, public services, legal supervision, and corruption control. New institutional economics suggests that under different institutional environments, companies will receive different investment returns [31]. [32] Also pointed out that the same transaction that an enterprise conducts in different countries may incur different costs due to institutional differences. The operating performance of an enterprise not only depends on its own management level but is also affected by the institutional environment where it operates. As part of the host country’s institutional environment, the governance capacity of the government will be transmitted to the acquirer through the acquired company, thus having a significant impact on the acquirer’s performance. Specific assumptions regarding governmental governance capacity and M&A performance are as follows.

### 3.1. Governmental governance capacity and short-term M&A performance

Chinese companies’ FDI has significant resource-seeking characteristics [33, 34]. In the short term, for the purpose of seeking resources, the acquirers go to countries with low governance capacity, which includes backward rule of law and serious corruption, to carry out M&A. Such M&A will have higher excess returns in the short term due to the acquisition of large amounts of overseas resources. In addition, when a company enters an unfamiliar host country to carry out M&A activities, there is a large information asymmetry with the target company in terms of laws, markets, and the company’s operating status, which may lead to unfavorable consequences such as improper decision-making, excessive payment of consideration, or political risk. Further, the level of corruption control in a country is positively related to the cost of information acquisition [35]. In this context, when a country’s institution is imperfect, the acquirer can obtain favorable information and convenience from the government by using corruption and other special methods at a lower cost, and then use the information advantage to take a favorable position in the negotiations of M&A and obtain positive M&A performance in a short time. However, this effect is not durable. In the long term, relying on special methods such as bribery to obtain information will increase the operating costs of the acquirer, and the marginal utility of obtaining convenience through it will diminish quickly. Therefore, we assume the following:

H1: The governance capacity of the host government has a restraining effect on the short-term M&A performance. The worse the governance capacity, the better the short-term M&A performance.

### 3.2. Governmental governance capacity and long-term M&A performance

First, based on the theory of transaction costs, transaction costs include the costs of pre-information search and negotiation with counterparties during the event and post surveillance, etc. The host country’s unsound legal system and unstable political system may make the contract signed by the acquirer unable to be bound or the legal property deprived of, which will invisibly increase the risks and operating costs of M&A [36]. Conversely, when the acquirer enters a host country with strong governance capacity to invest, the higher efficiency of the government can not only simplify the approval procedures for M&A but also reduce the unnecessary losses caused by enterprises solving cumbersome administrative problems in the operation after the completion of M&A, thus laying a good foundation for the long-term operation of the acquirer.

Secondly, based on the theory of information asymmetry, the information asymmetry between the acquirer and the target is an important factor affecting the value creation of M&A [37, 38]. According to the theory of liability of foreignness, due to the differences in politics, economy, culture, institution, and other aspects between the home country and the host country, it will lead to the lack of legitimacy of the acquirer and the problem of information asymmetry [39, 40]. On the one hand, in the early stage of M&A, the acquirer’s poor understanding of the accounting standards and tax policies of a host country where the target company is located may lead to inaccurate asset evaluations and affect M&A performance [41]. On the other hand, in the follow-up business process, the stakeholders in the host country lack information about the background or brand of the transnational acquirer, which increases the unfamiliar cost, and they may therefore give up cooperation with the acquirer [42], thus making it difficult to guarantee the long-term performance of the M&A. When the acquirer enters a host country with strong governance capacity to carry out M&A activities, the open and transparent investment environment created by the government is conducive to enhancing the acquirer’s understanding of the host country’s relevant policies and regulations, ensuring the transparency of market transactions, and reducing the acquiring party’s information search costs and information uncertainty, which is helpful in improving the accuracy of the target company’s asset evaluation. Moreover, a good government regulatory system can urge all parties to make complete and true information disclosures to ensure market transparency and reduce information asymmetry between the parties to the transaction, which not only avoids business risks for the acquirer and provides good protection for its shareholders’ long-term interests but also helps to enhance the awareness of the host country stakeholders of foreign enterprises and reduce the disadvantages brought about by transnational acquirers as outsiders, thus contributing to the improvement of the long-term performance of the acquirer.

Finally, based on the theory of institutional isomorphism, in a certain organizational field, the legality of the system requires that corporate behaviors are consistent with the rule of law, social regulations, and other institutional factors. This powerful force from institutional norms will tend to make the characteristics and behaviors of enterprises in the same field consistent. Acquirers will also face the pressure of institutional isomorphism in the process of entering the host country to carry out operations, and they have the motivation to actively imitate the behavior of local enterprises in the host country to achieve institutional convergence in order to gain legitimacy and overcome the disadvantages of outsiders [43]. In countries with sound legal systems, the government has made clear provisions on the construction of internal control and related disclosure requirements of enterprises. Correspondingly, under this institutional environment, the company has a complete corporate governance structure and effective internal control mechanism, which is conducive to reducing the cost of corporate equity and bond financing to enhance corporate value [44]. When entering a country with strong governance capacity to carry out M&A activities, due to the pressure of the host country’s sound system, the acquirer can effectively improve the internal governance structure of the company after the merger, and consciously imitate the relevant norms of successful companies to compensate for the disadvantage of outsiders caused by the lack of legitimacy and show better M&A performance [45]. Therefore, we assume the following:

H2: The governance capacity of the host government has a positive effect on the long-term M&A performance. The better the governance capacity, the better the long-term M&A performance.

## 4. Research design

### 4.1. Sample selection

We selected completed cross-border M&A transactions of China’s Shanghai and Shenzhen listed companies from 2007 to 2018 included in the Wind Financial Terminal as the initial sample, and selected them according to the following criteria: (1) Remove the related M&A transactions between the acquirer and its overseas holding company; (2) Remove the M&A transactions where the target company is registered in the Cayman Islands, the Virgin Islands, Bermuda, or other tax havens; (3) Remove M&A transactions involving two or more countries; (4) Exclude M&A cases where the total price of M&A transactions is not disclosed in the announcement; (5) Exclude M&A cases in which the total price of M&A transactions accounted for less than 0.5% of the market value, sales revenue, and total assets of the acquirer in the year of M&A disclosure. Through the above criteria screening, we finally obtained 460 cross-border M&A transactions as empirical samples.

### 4.2. Empirical model

According to the above hypothesis, to test the relationship between the host country’s governmental governance capacity and M&A performance, we constructed the following empirical analysis model:

$$CAR=\alpha_{0}+\alpha_{1}{WGI}_{i}+\alpha_{2-11}\sum controls+\varepsilon ,$$ (1)

$$BHAR=\beta_{0}+\beta_{1}{WGI}_{i}+\beta_{2-11}\sum controls+\varepsilon ,$$ (2)

where *CAR* and *BHAR* are the dependent variables, representing the short-term and long-term cross-border M&A performance, respectively; *WGI* is the main independent variable, representing the six-dimensional WGI index; and *controls* represent control variables. Since each WGI dimension index is highly collinear, we averaged the six-dimensional indexes to observe the influence of a country’s overall governmental governance capacity on the cross-border M&A performance of the acquirer. At the same time, we returned to each dimension index separately to examine the influence of different dimensions of governance capacity on the long-term performance of cross-border M&A.

### 4.3. Research variables

#### 4.3.1. Dependent variables

The dependent variables are the short-term and long-term M&A performance of the acquirer. The measurement of M&A performance includes two types: accounting research method and event research method. The accounting research method is vulnerable to earnings management by financial statement preparers, which affects the credibility of financial data, while stock price fluctuation reflects historical information such as the operation of listed companies clearly, so we used the event research method to measure M&A performance.

Short-term M&A performance is measured by the cumulative excess return (CAR), referring to the market model of [46], that is, *R*_*it*_ = *α*_*i*_ + *β*_*i*_*R*_*mt*_ + *ε*_*it*_ · *R*_*it*_ and *R*_*mt*_ are the daily return rate of individual stocks and the daily return rate of market, respectively. We took 150 trading days to 30 trading days before the first disclosure date of an M&A transaction as the estimation period, and then calculated the cumulative excess rate of return based on the cumulative difference between the expected rate of return during the event period calculated by the estimation model and the actual rate of return of the company. The event window periods were [–3,3] and [–5,5], and the relevant original data were taken from the CSMAR database.

For long-term M&A performance, we used the purchase and hold abnormal rate of return (BHAR) for measurement. The observation periods were 12 months and 24 months. The calculation formula is

$${BHAR}_{i,t}=\prod_{t=0}^{T}\left(1+R_{it}\right)-\prod_{t=0}^{T}(1+R_{pt}),$$ (3)

where T is divided into two groups of 0–24 and 0–36, t = 0 denotes the month of the merger announcement date, t = 1 denotes one month after the merger announcement date, and so on. *R*_*it*_ represents the actual monthly return rate of stock i in month t, and *R*_*pt*_ represents the monthly return rate of the corresponding portfolio of stock i in month t. For the monthly return rate of the corresponding portfolio, refer to the method of [47]. The relevant raw data were accessed using the CSMAR database.

#### 4.3.2. Independent variables

The main independent variable is the governance capacity of the host country government. Regarding governmental governance capacity, we referred to the method of [48], and selected the WGI compiled by the World Bank to measure it. The WGI describe the governance capacity of a country’s government based on six dimensions, including voice and accountability (*voice*), political stability (*polit*), government effectiveness (*govern*), regulatory quality (*regul*), rule of law (*law*), and control of corruption (*corrupt*). The value of each dimension index ranges from -2.5 to 2.5. The higher the score, the better the governance capacity. Since the six dimensions of the WGI are not independent of each other, we averaged the six dimensions of the index to observe the impact of the host country’s overall governance capacity (*average*) on the M&A performance. Furthermore, we analyzed the different effects of six dimensions on the long-term M&A performance of the acquirer and discussed the areas that the acquirer should focus on in the process of cross-border M&A.

#### 4.3.3. Control variables

Based on the existing research on M&A performance and foreign investment, we selected indicators that could reflect the situation of the host country, the characteristics of the acquirer, and the relationship between the host country and the home country as the control variables. Among them, the host country’s gross domestic product (*gpd*) and economic freedom index (*freedom*) reflected the situation of the host country; the total assets of the acquirer (*lnasset*), the asset-liability ratio (*leverage*), operating cash flow (*ocf*), equity concentration (*top1*), and transaction scale (*scale*) reflect the characteristics of the acquirer; the import and export trade volume (*lntrade*), the geographic distance between the two parties (*lndist*), and the degree of language similarity between the two parties (*langua*) reflect the relationship between the host country and the home country.

## 5. Empirical results and analysis

### 5.1. Descriptive statistics

Table 1 shows the descriptive statistics of the variables. It can be seen from the table that the average value of short-term M&A performance CAR[–3,3] and CAR[-5.5] are positive, indicating that the market is optimistic about corporate cross-border M&A. However, the long-term M&A performance of the acquirer is poor, with the mean BHAR of 24 months and 36 months after M&A being -0.0714 and -0.124, respectively, indicating that cross-border M&A will cause acquirers value impairment in the long term. In addition, the standard error of each independent variable is in the range of 0.614 to 0.863, which indicates that the governance level of governments in different countries varies.

**Table 1:** Descriptive statistics.

Variable	Obs	Mean	Std. Dev	Min	Max
*CAR[-3*.*3]*	460	0.0218	0.126	-0.548	0.501
*CAR[–5*,*5]*	460	0.0172	0.163	-0.597	0.848
*BHAR24*	356	-0.0714	0.858	-2.225	8.364
*BHAR36*	289	-0.124	0.976	-2.743	7.152
*average*	459	1.123	0.628	-1.667	1.860
*voice*	356	0.963	0.635	-1.723	1.688
*polit*	356	0.576	0.614	-2.351	1.615
*govern*	356	1.326	0.671	-1.635	2.437
*regul*	356	1.329	0.664	-1.466	2.261
*law*	356	1.301	0.746	-1.690	2.046
*corrupt*	356	1.251	0.863	-1.418	2.381
*freedom*	460	73.40	8.221	40.60	89.40
*gdp*	460	51,789	71,552	8.319	205,802
*lnasset*	460	13.61	1.697	10.38	21.45
*leverage*	460	0.447	0.195	0.0246	0.939
*ocf*	460	0.0488	0.0622	-0.153	0.291
*top1*	460	0.339	0.147	0.0696	0.894
*scale*	460	0.819	11.52	0.000247	239.8
*lntrade*	459	6.713	1.621	-0.440	8.795
*lndist*	457	8.928	0.572	6.862	9.868
*langua*	456	0.363	0.164	0	0.713

### 5.2. Empirical regression results and analysis

#### 5.2.1. Governmental governance capacity and short-term and long-term M&A performance

Based on empirical models (1) and (2), the data used in this empirical study are discrete cross-sectional data; thus, OLS regression with robust standard errors was used to control possible heteroscedasticity problems. The regression results are shown in Table 2. Among them, columns (1,2) are listed as the impact of governmental governance capacity on short-term M&A performance, and columns (3,4) are listed as the impact on long-term M&A performance.

**Table 2:** Governmental governance capacity and short-term and long-term M&A performance.

	Short-term M&A performance		Long-term M&A performance	
Variable	(1)	(2)	(3)	(4)
	CAR[–3,3]	CAR[–5,5]	BHAR24	BHAR36
*average*	-0.033*	-0.053**	0.291**	0.297*
	(-1.71)	(-1.97)	(2.00)	(1.76)
*freedom*	0.001	0.001	-0.027**	-0.019
	(0.54)	(0.72)	(-2.07)	(-1.30)
*gdp*	-0.000	-0.000*	-0.000	-0.000
	(-1.26)	(-1.70)	(-1.03)	(-0.42)
*lnasset*	-0.001	-0.002	-0.036	-0.033
	(-0.26)	(-0.42)	(-1.22)	(-0.94)
*leverage*	0.021	0.040	-0.006	0.252
	(0.50)	(0.70)	(-0.02)	(0.66)
*ocf*	-0.014	-0.076	1.127	1.817*
	(-0.15)	(-0.70)	(1.16)	(1.71)
*top1*	0.031	0.064	-0.426	-0.483
	(0.70)	(1.14)	(-1.31)	(-1.31)
*scale*	-0.000	-0.000**	-0.000	0.003
	(-0.45)	(-2.38)	(-0.06)	(0.78)
*lntrade*	-0.001	0.001	0.067	0.038
	(-0.16)	(0.10)	(1.25)	(0.60)
*lndist*	0.006	0.020	0.037	-0.043
	(0.47)	(1.21)	(0.43)	(-0.42)
*langua*	0.017	0.003	-0.025	0.424
	(0.39)	(0.06)	(-0.08)	(1.10)
*constant*	-0.044	-0.187	1.498	1.354
	(-0.29)	(-0.96)	(1.58)	(1.28)
N	454	454	352	286

T-values are in parenthesis.

***, **, and * denote significance at the 1%, 5%, and 10% levels, respectively.

According to the empirical results, the following conclusions can be drawn: (1) The regression coefficients of governmental governance capability (*average*) and CAR[–3,3] and CAR[–5,5] are significantly negative at the 10% and 5% level, respectively. That is, in the short term, selecting the host country with poor governance capacity for M&A activities can achieve excess returns and provide a short-term boost to M&A performance. H1 was established. For the purpose of seeking resources, many Chinese companies select countries with poor governmental governance capacity to carry out M&A activities. Such M&A have enabled the acquirer to obtain a large number of scarce resources in a short period of time, thereby driving the growth of the acquirer’s short-term performance. (2) However, the regression coefficients of governmental governance capability (*average*) and BHAR24 and BHAR36 are significantly positive, which indicates that weak governmental governance can inhibit M&A performance in the long term. On the contrary, strong governmental governance ability will promote the improvement of the long-term comprehensive performance of the acquirer. The better the governance ability, the higher the value appreciation of the acquirer in the long term. H2 is thus established. (3) Generally speaking, the purpose of the existence of an enterprise is to obtain sustainable added value to maximize the interests of shareholders. In the process of cross-border M&A, although companies seeking resources to conduct M&A in countries with poor governmental governance capacity can obtain short-term excess returns, the effect is not sustainable. In the long term, such M&A will ultimately result in corporate value derogation. Consequently, in order to obtain long-term stable value growth, when arranging overseas M&A, the acquirer should avoid short-sighted behavior of going to countries with low governance capacity in pursuit of short-term benefits, and focus on the impact of governmental governance capacity on the long-term interests of the acquirer.

#### 5.2.2. Six dimensions of governmental governance capacity and long-term M&A performance

From the above analysis, it can be seen that in order to maximize shareholder value, the acquirer should focus on the long-term influence of governance capacity on M&A when deploying overseas M&A, and consciously lean toward countries with strong governance capacity of government when making cross-border M&A transaction decisions. However, governance capacity of government is an overall concept. According to the WGI compiled by the World Bank, it can be divided into six aspects, as mentioned above. There are also big differences in the specific dimension indexes between countries, so which dimensions have the deepest impact on long-term M&A performance? What factors should the acquirer focus on when arranging overseas M&A activities? The following section will conduct regression analysis on the influence of the six dimensions of the WGI on long-term M&A performance, exploring the areas that the acquirer should focus on in the process of cross-border M&A.

Tables 3 and 4 shows the regression results of the six specific dimensions of governance capacity and long-term M&A performance. Among them, Table 3 are listed as regression results of governance capacity and BHAR24, and Table 4 are regression results of BHAR36. According to the empirical results, the following conclusions can be drawn: (1) Voice and accountability (*voice*) and political stability (*polit*) have no significant impact on the long-term M&A performance of acquirers. The development of democratic politics in the host country and a stable business environment cannot bring long-term added value to the acquirer. (2) The government effectiveness (*govern*) coefficient is significantly positive at the 5% level. High-quality and efficient government services are conducive to the improvement of the acquirer’s long-term M&A performance. A possible reason is that the high government efficiency can not only simplify M&A approval procedures, in the operation after the completion of M&A, but also reduce unnecessary losses caused by complicated administrative problems, lowering transaction costs. Moreover, the high-quality public services and infrastructure provided by the effective government enable the acquirer to obtain a number of innovative resources, policy support, and perfect market network fairly and effectively through the market mechanism, thus laying a foundation for the long-term operation of the acquirer. (3) Regulatory quality (*regul*) coefficient is significantly positive at the 1% level, which means government supervision can promote M&A performance in the long term. A possible reason is that a good government control system can urge all parties to make complete and true information disclosures to ensure market transparency, reduce information asymmetry with the counterparty, and provide good protection for shareholders’ long-term interests. (4) Rule of law (*law*) and control of corruption (*corrupt*) have a positive impact on the long-term M&A performance of acquirers, and both are significant at the 10% level. A sound rule of law system and a strong level of corruption control are beneficial to standardizing the trading behavior of various subjects in the market and promoting fair trade, thus improving the investment efficiency and performance of acquirers. (5) On the whole, the government effectiveness, regulatory quality, rule of law, and control of corruption all have a significant positive impact on long-term M&A performance. However, from the perspective of the degree of influence, the regression coefficient of government effectiveness and regulatory quality is the largest in absolute value and most significant. Obviously, management should pay special attention to the efficiency of the host country’s government and the quality of regulation when deploying overseas M&A. The government efficiency and high-quality regulatory quality of the host country can effectively reduce transaction costs, alleviate the degree of enterprise information asymmetry, overcome the disadvantages of outsiders of the acquirer to a certain extent, and ultimately contribute to the improvement of long-term M&A performance. Furthermore, the regression coefficient of government effectiveness and BHAR36 (0.395) is larger than that of BHAR24 (0.299). Similarly, the regression coefficient of regulatory quality and BHAR36 (0.600) is also greater than that of BHAR24 (0.449), which shows that, over time, the influence of government effectiveness and regulatory quality on M&A performance becomes increasingly obvious, and the influence of governance capacity on M&A performance will play a more prominent role over a longer period of time.

**Table 3:** Six dimensions of governmental governance capacity and BHAR24.

	(1)	(2)	(3)	(4)	(5)	(6)
	BHAR24	BHAR 24	BHAR 24	BHAR 24	BHAR 24	BHAR 24
*voice*	0.120*					
	(1.73)					
*polit*		0.144				
		(1.26)				
*govern*			0.299**			
			(2.15)			
*regul*				0.449***		
				(2.96)		
*law*					0.197*	
					(1.71)	
*corrupt*						0.219*
						(1.76)
*freedom*	-0.012*	-0.017	-0.029**	-0.040***	-0.023*	-0.028*
	(-1.71)	(-1.54)	(-2.20)	(-2.82)	(-1.86)	(-1.93)
*gdp*	-0.000	-0.000	-0.000	-0.000	-0.000	-0.000
	(-0.96)	(-1.01)	(-1.17)	(-0.88)	(-1.18)	(-0.95)
*lnasset*	-0.035	-0.033	-0.038	-0.034	-0.036	-0.038
	(-1.21)	(-1.14)	(-1.31)	(-1.15)	(-1.24)	(-1.30)
*leverage*	-0.021	-0.072	0.013	0.021	-0.008	-0.006
	(-0.07)	(-0.22)	(0.04)	(0.07)	(-0.02)	(-0.02)
*ocf*	1.089	1.069	1.093	1.052	1.077	1.069
	(1.10)	(1.09)	(1.12)	(1.07)	(1.10)	(1.10)
*top1*	-0.427	-0.410	-0.424	-0.440	-0.421	-0.411
	(-1.31)	(-1.27)	(-1.31)	(-1.36)	(-1.30)	(-1.28)
*scale*	0.000	-0.000	-0.000	0.000	-0.000	0.000
	(0.09)	(-0.04)	(-0.12)	(0.16)	(-0.05)	(0.09)
*lntrade*	0.058	0.076	0.065	0.054	0.068	0.067
	(1.12)	(1.31)	(1.23)	(1.07)	(1.26)	(1.23)
*lndist*	0.030	0.069	0.067	0.031	0.051	0.008
	(0.35)	(0.75)	(0.76)	(0.35)	(0.59)	(0.10)
*langua*	-0.003	-0.016	0.046	-0.011	0.070	0.079
	(-0.01)	(-0.05)	(0.15)	(-0.03)	(0.22)	(0.25)
*constant*	0.679	0.645	1.286	2.204**	1.092	1.802
	(0.90)	(0.81)	(1.48)	(2.20)	(1.28)	(1.61)
N	352	352	352	352	352	352

T-values are in parenthesis.

***, **, and * denote significance at the 1%, 5%, and 10% levels, respectively.

**Table 4:** Six dimensions of governmental governance capacity and BHAR36.

	(1)	(2)	(3)	(4)	(5)	(6)
	BHAR 36	BHAR 36	BHAR 36	BHAR 36	BHAR 36	BHAR 36
*voice*	0.074					
	(0.84)					
*polit*		0.022				
		(0.16)				
*govern*			0.395**			
			(2.26)			
*regul*				0.600***		
				(2.92)		
*law*					0.223*	
					(1.74)	
*corrupt*						0.243*
						(1.80)
*freedom*	-0.002	-0.001	-0.027*	-0.040**	-0.016	-0.021
	(-0.25)	(-0.10)	(-1.76)	(-2.34)	(-1.23)	(-1.42)
*gdp*	-0.000	-0.000	-0.000	-0.000	-0.000	-0.000
	(-0.37)	(-0.40)	(-0.59)	(-0.23)	(-0.61)	(-0.33)
*lnasset*	-0.034	-0.034	-0.035	-0.028	-0.034	-0.036
	(-1.00)	(-1.00)	(-1.03)	(-0.81)	(-0.99)	(-1.05)
*leverage*	0.229	0.212	0.309	0.300	0.270	0.265
	(0.60)	(0.53)	(0.82)	(0.79)	(0.71)	(0.70)
*ocf*	1.746	1.696	1.799*	1.800*	1.788*	1.768*
	(1.62)	(1.58)	(1.69)	(1.68)	(1.67)	(1.66)
*top1*	-0.470	-0.454	-0.495	-0.522	-0.482	-0.461
	(-1.28)	(-1.26)	(-1.35)	(-1.43)	(-1.31)	(-1.28)
*scale*	0.003	0.003	0.002	0.003	0.003	0.003
	(0.84)	(0.85)	(0.77)	(0.81)	(0.80)	(0.79)
*lntrade*	0.027	0.031	0.041	0.029	0.040	0.036
	(0.44)	(0.46)	(0.65)	(0.48)	(0.62)	(0.58)
*lndist*	-0.035	-0.016	-0.006	-0.055	-0.030	-0.075
	(-0.35)	(-0.15)	(-0.06)	(-0.54)	(-0.30)	(-0.74)
*langua*	0.460	0.517	0.497	0.436	0.516	0.530
	(1.16)	(1.30)	(1.32)	(1.13)	(1.37)	(1.40)
*constant*	0.390	0.193	1.387	2.546**	1.067	1.837
	(0.45)	(0.20)	(1.37)	(2.14)	(1.10)	(1.51)
N	286	286	286	286	286	286

T-values are in parenthesis.

***, **, and * denote significance at the 1%, 5%, and 10% levels, respectively.

#### 5.2.3. Robustness test

We used CAR[–3,3] and CAR[–5,5] as short-term M&A performance measurement indicators, and used BHAR24 and BHAR36 as long-term M&A performance measurement indicators. The regression results of CAR[–3,3] and CAR[–5,5] and governance capacity of government are basically the same, and so was BHAR, which demonstrates that the empirical regression conclusion is to an extent robust. In addition, we also considered the method of replacing indicators for robustness test. For short-term M&A performance, we used CAR[–10,10], which is the cumulative excess return 10 days before and after the M&A disclosure date to replace the original CAR[–3,3] and CAR[–5,5] for robustness test; for long-term M&A performance, we used Δ*ROE*_*t*−3,*t*+3_, which is the difference in the return on net assets of the three years before and after the disclosure year of the merger to replace the original BHAR24 and BHAR36 for robustness test. The robustness results are shown in Tables 5 and 6. Table 5 shows the test results of the overall governance capacity of government and short-term and long-term M&A performance after replacing the indicators. The research results are consistent with the above. M&A activities carried out in countries with lower governance capacity can obtain excess returns in the short term, but in the long term it will damage the equity of shareholders. Conversely, M&A in countries with strong governmental governance capacity can enable acquirers to obtain higher value appreciation in the long term. Table 6 shows the robustness test results of the six dimensions of governance capacity and long-term M&A performance. It can be seen that, although control of corruption is no longer robust, government effectiveness, regulatory quality, and rule of law still significantly promote the improvement of long-term M&A performance of acquirers. Consequently, in order to improve the success rate of cross-border M&A, companies should pay attention to the efficiency, regulatory control, and rule of law of the host country’s government when deploying overseas M&A.

**Table 5:** Robustness test of governance capacity and short-term and long-term M&A performance.

	Short-term M&A performance	Long-term M&A performance
	(1)	(2)
	CAR[–10,10]	Δ*ROE*_*t*−3,*t*+3_
*average*	-0.072**	0.079*
	(-2.56)	(1.75)
*freedom*	0.007***	-0.007*
	(2.93)	(-1.92)
*gdp*	-0.000	0.000
	(-0.06)	(0.67)
*lnasset*	-0.001	0.009
	(-0.08)	(0.78)
*leverage*	0.008	0.149
	(0.12)	(1.49)
*ocf*	-0.053	0.297
	(-0.32)	(1.15)
*top1*	0.029	-0.043
	(0.43)	(-0.43)
*scale*	0.000	0.000
	(0.48)	(0.43)
*lntrade*	-0.012	0.011
	(-1.13)	(0.62)
*lndist*	-0.002	-0.012
	(-0.10)	(-0.36)
*langua*	0.080	-0.026
	(1.10)	(-0.23)
*constant*	-0.332	0.130
	(-1.28)	(0.33)
N	425	290

T-values are in parenthesis.

***, **, and * denote significance at the 1%, 5%, and 10% levels, respectively.

**Table 6:** Robustness test of six dimensions of governance capacity and long-term M&A performance.

	(1)	(2)	(3)	(4)	(5)	(6)
	Δ*ROE*_*t*−3,*t*+3_	Δ*ROE*_*t*−3,*t*+3_	Δ*ROE*_*t*−3,*t*+3_	Δ*ROE*_*t*−3,*t*+3_	Δ*ROE*_*t*−3,*t*+3_	Δ*ROE*_*t*−3,*t*+3_
*voice*	0.049*					
	(1.71)					
*polit*		-0.003				
		(-0.06)				
*govern*			0.091*			
			(1.89)			
*regul*				0.114**		
				(2.15)		
*law*					0.068*	
					(1.81)	
*corrupt*						0.053
						(1.58)
*freedom*	-0.003	-0.002	-0.008**	-0.009**	-0.006*	-0.006*
	(-1.30)	(-0.50)	(-2.05)	(-2.29)	(-1.95)	(-1.79)
*gdp*	0.000	0.000	0.000	0.000	0.000	0.000
	(0.72)	(0.53)	(0.54)	(0.98)	(0.38)	(0.70)
*lnasset*	0.009	0.008	0.008	0.010	0.008	0.007
	(0.77)	(0.71)	(0.73)	(0.89)	(0.76)	(0.68)
*leverage*	0.150	0.138	0.154	0.144	0.152	0.151
	(1.50)	(1.38)	(1.54)	(1.45)	(1.52)	(1.51)
*ocf*	0.301	0.250	0.293	0.299	0.295	0.278
	(1.17)	(0.97)	(1.14)	(1.17)	(1.15)	(1.08)
*top1*	-0.045	-0.032	-0.046	-0.052	-0.041	-0.035
	(-0.46)	(-0.33)	(-0.47)	(-0.53)	(-0.42)	(-0.36)
*scale*	0.000	0.000	0.000	0.000	0.000	0.000
	(0.45)	(0.47)	(0.42)	(0.46)	(0.43)	(0.46)
*lntrade*	0.008	0.011	0.011	0.007	0.012	0.011
	(0.45)	(0.63)	(0.61)	(0.41)	(0.66)	(0.65)
*lndist*	-0.018	-0.000	-0.002	-0.014	-0.010	-0.016
	(-0.50)	(-0.00)	(-0.07)	(-0.40)	(-0.28)	(-0.45)
*langua*	-0.046	-0.011	-0.005	-0.005	-0.003	-0.003
	(-0.40)	(-0.09)	(-0.04)	(-0.04)	(-0.03)	(-0.02)
*constant*	-0.032	-0.245	0.097	0.267	0.092	0.157
	(-0.09)	(-0.67)	(0.25)	(0.65)	(0.24)	(0.38)
N	290	290	290	290	290	290

T-values are in parenthesis.

***, **, and * denote significance at the 1%, 5%, and 10% levels, respectively.

## 6. Conclusions

In the context of globalization, Chinese companies have become deeply integrated into the world economy, and the number of cross-border M&A transactions has grown rapidly. However, only a few acquirers can achieve their strategic goals, and most M&A activities do not add value to companies. Existing documents have provided some explanations of this problem from the perspective of enterprises, transactions, and macroeconomics. As an important part of the macroeconomic environment, the host country system is rarely analyzed, especially from the perspective of governance capacity of government. Based on this, we selected the cross-border M&A cases of China’s Shanghai and Shenzhen listed companies from 2007 to 2018 as research samples and built an empirical model to test the relationship between the host country’s governmental governance capacity and M&A performance of acquirers. It was found that the governance capacity of government has an inhibitory effect on cross-border M&A performance in the short term but can promote its improvement in the long term. Specific to the six different dimensions of governance capacity on long-term M&A performance, the impact of government effectiveness, regulatory quality, and rule of law are the most significant.

The above conclusions have certain reference value for the practical activities of Chinese companies. Although in the short term, for the purpose of seeking resources, the acquirer goes to some countries with loose regulation, backward rule of law, and serious corruption to carry out M&A, which can secure a large amount of energy and mineral resources and have higher returns. However, in the long term, ignoring the negative impact of weak governance capacity will increase the risk for acquirers, and ultimately result in a loss of corporate value. Conversely, M&A activities in countries with strong governance capacity can enable companies to obtain higher added value in the long term. Consequently, for the sustainable development of business operations and the maximization of shareholder value after M&A, the acquirer should pay more attention to the long-term impact of governance capacity when deploying overseas M&A, and consciously lean toward countries with strong governance capacity when making cross-border M&A transaction decisions. At the same time, the acquirer should consider the long-term risks of M&A, so as to make M&A as consistent with the long-term development goals of enterprises as possible.

The research deficiencies are mainly reflected in the following aspects: First, sample selection bias. In the existing research on Chinese companies’ cross-border M&A, different scholars have selected different database samples and different screening criteria, and the final conclusions drawn may be inconsistent due to sample selection errors. At the same time, affected by the small number of M&A cases of Chinese listed companies, and we excluded M&A cases where the target company is registered in tax havens such as the Cayman Islands or the M&A transaction price is too low, finally 460 cross-border M&A transactions are obtained as the empirical sample, with few sample cases, and have a certain impact on the conclusion. Second, we failed to further empirically explore the non-linear effect of governmental governance capabilities on M&A performance. We only focused on the linear impact of governmental governance capabilities on short-term and long-term M&A performance. In future research, we can further explore the non-linear effects of governmental governance capabilities.

## Supporting information

S1 File Dataset. (XLSX)
